# Insights from assessment for arrhythmia after CoViD-19 vaccination among Indians

**DOI:** 10.6026/973206300200430

**Published:** 2024-05-31

**Authors:** S Sunita, Madhu Bala Negi, Moti Lal, Chandan Kumar, Manish Kumar

**Affiliations:** 1 Department of Physiology, Indira Gandhi Institute of Medical Sciences, Patna, Bihar, India; 2 Department of Medicine, ABVIMS, New Delhi, India; 3 Lady Hardinge Medical College, New Delhi, India

**Keywords:** ECG, Covieshield vaccine, myocarditis, arrhythmia

## Abstract

Post-vaccination myocarditis is usually moderate and transient, recovering quickly with conservative treatment. Therefore, it is of interest to assess for arrhythmia after CoViD-19 vaccination among Indians. We looked for ECG abnormalities in a small cohort of 50 participants after 52 weeks after receiving the Oxford/AstraZeneca CoViD-19 vaccination. Data shows that post-vaccination myocarditis is typically mild and transient, with most cases resolving swiftly through conservative management. Thus, it is unlikely that this vaccine will induce severe arrhythmias or life-threatening cardiac events in the general population.

## Background:

The global CoViD-19 epidemic has caused over 660 million illnesses and over 6.7 million deaths as of January 2023 [1]. The main weapon in the fight to stop this epidemic is vaccination. The CoViD-19 pandemic's full impacts were felt in India. The Oxford-Astra Zeneca vaccine, manufactured under license by Serum Institute of India and sold as Covishield, was the first vaccination to receive emergency use authorization in India for the prevention of Covid-19 infection [1]. The effectiveness and safety of CoViD-19 immunizations in preventing major CoViD- 19 episodes have been shown by randomized controlled trials. During these trials, headaches, nausea, vomiting, diarrhea, and myalgia (muscle pain) were among the most frequent adverse effects reported by participants [2, 3]. There have been cases of suspected myocarditis following immunization, even though myocarditis was not noted as a side effect in clinical trials [4,5]. Myocarditis can present with a wide variety of symptoms, from modest trouble breathing or chest pain that goes away on its own to more serious effects including cardiogenic shock or sudden death [6]. This could have led to undetected cases of myocarditis following immunization. Defects in the studies could be the reason why the CoViD-19 immunization clinical trials were unable to identify uncommon side effects [4]. It is well-recognized that myocarditis can result in extensive or localized heart muscle scarring or fibrosis. This can disrupt the heart's regular electrical activity and create an abnormal conduction pathway. The circumstances for re-entry events and subsequent arrhythmias are produced by this altered path [7]. Therefore, it is of interest to assess abnormalities in the electrocardiogram (ECG) patterns of individuals who were given a precautionary dosage (which was administered 52 weeks following the Oxford/AstraZeneca CoVid-19 vaccination).

## Materials and Method:

The purpose was to analyze ECG changes in interval, length, and amplitude after 52 weeks following immunization. The study started on March 16th and ended on March 30th, 2022. 70 individuals over the age of 60 years were recruited for the study. These participants visited the Community Medicine OPD of IGIMS, Patna to receive a precautionary dose, which was delivered 52 weeks after the initial dosage. After history taking, 14 were excluded based on previous SARS-CoV-2 infection, hypertension, diabetes mellitus, cardio-respiratory disease, thyroid disorder, or medication that could impact sinus rhythm. Data from 6 people were excluded from the analysis due to signal artefacts in ECG recordings. Thus, 20 participants were excluded. The final analysis was performed on data obtained from 50 patients (23 male, 27 female).

## Anthropometry:

The research participants were subjected to assessments of their height and weight, and their body mass index (BMI) was computed using the gathered data. The measurements were carried out by a sole investigator with prior anthropometry competence. A female attendant was present to assist and prepare female participants for height and weight measurement. The height was assessed using a portable stadiometer (Precision Model, Prime Surgical, New Delhi, India) with an accuracy of 1 mm, while weight was measured in kilograms.

## Electrocardiogram recording:

Initial measurements of resting heart rate (RHR), systolic blood pressure (SBP), and diastolic blood pressure (DBP) were performed. In addition, we collected measurements for RR, PR, QRS, QT, QTc, JT intervals, and T peak-T end intervals, as well as the amplitudes of P, Q, R, S, and T waves. The data acquisition was performed using a 4-channel Power Lab15 T (Model ML 818) and analyzed using LAB Chart Pro software version: v8.1.13 (AD Instrument Ltd. Australia). The Power Lab data acquisition equipment effectively identified and transformed analogue ECG impulses into digital data without any interruption. In addition, the ECG Analysis Module of the LAB Chart Pro program enabled the recognition of Lead II ECG waveforms and automatically detected heart rhythm, amplitude, and the initiation of P, Q, R, S, and T w.

## Results:

The data from a total of 23 males and 27 females were analyzed, with results expressed as mean (standard errors). Table 1 displays the anthropometric parameters of the participants. Table 2 presents the hemodynamic parameters and ECG intervals. Additionally, ECG wave amplitudes were measured and found to be within normal limits for both males and females. (Table 3) [8]

## Statistical analysis:

ta obtained was analysed using Excel software and SPSS version 22 to find the mean and standard deviation of parameters in all subjects.

## Discussion:

Instances of post-vaccination myocarditis have been documented in the United States, United Kingdom, and Israel, and a small number of cases have also been reported in India [9,10, 11,12]. Diagnosing acute myocarditis is difficult due to the absence of a specific clinical presentation and the potential for confusion with several other non-inflammatory cardiac illnesses, such as acute coronary syndrome (ACS) [6]. Moreover, myocarditis can result in serious outcomes, including undetected heart problems that may only become apparent several months after a person has recuperated from COVID-19, potentially leading to heart failure [13]. If fibrosis or scarring occurs in the myocardium, it can impede the conduction route and result in abnormal ECG patterns and re-entrant arrhythmias in cases of myocarditis. The presence of fibrosis or scarring in the myocardium can lead to specific alterations in the electrocardiogram (ECG). These changes include lengthening of the QRS complex, deep Q waves, T wave inversions, and fragmentation of the QRS complex. These ECG findings are all diagnostic of the presence of myocardial fibrosis [14].

In India, there is a shortage of studies examining the possible enduring electrocardiogram (ECG) alterations in patients aged 60 and above after receiving the Oxford/AstraZeneca CoVID-19 vaccine. To fill this need, our research sought to investigate the possibility of ECG analysis as a non-invasive technique for the early identification of fibrotic cardiomyopathy [15]. This could potentially assist in the prevention of arrhythmias. The study utilized 4 channels of Power Lab for data collecting and the analysis was performed using the LAB Chart Pro software version. Analysed ECG data from a combined group of 23 males and 27 females were examined 52 weeks following vaccination [16]. The study noted that RR, PR, QRS, QT, QTc, JT, and T peak T end intervals did not show any signs of prolongation. In addition, the amplitudes of P, Q, R, S, and T were measured and determined to be within the normal range [17]. Significantly, there were no reported instances of QRS extension, deep Q waves, T wave inversions, or QRS fragmentation. Furthermore, the current study did not identify any cases of clinical myocarditis [18].

In our study no abnormalities in the electrocardiogram (ECG) after 52 weeks after the initial dose was detected. This reduces the possibility of life-threatening irregular heart rhythms in the population vaccinated with the Oxford/AstraZeneca CoVID-19 vaccine. These findings have important implications for the general population, clinicians, and policymakers. They provide reassurance regarding the vaccine's safety about cardiac health. Multiple studies have emphasized the heightened likelihood of myocarditis after receiving mRNA-based CoViD-19 vaccinations, especially among individuals of different age groups and genders. Significantly, the likelihood of risk is most pronounced among teenage males and young adult males after receiving the second dose of the vaccine [9]. Patone *et al.* conducted population-based research that identified links between rare cardiac effects and both the three CoViD-19 vaccinations and the SARS-CoV-2 infection. Their research uncovered those adults who received vaccinations against SARS-CoV-2 faced a heightened likelihood of developing myocarditis within one week of getting the initial dosage of both adenovirus and mRNA vaccines, as well as after receiving the second dose of mRNA vaccines [4]. These observations highlight the crucial importance of on-going monitoring and evaluation of possible negative consequences associated with CoViD-19 immunization. Monitoring is crucial for upholding public health and guaranteeing the safety of immunization programs. Data shows no evidence of an increased risk of myocarditis or cardiac arrhythmias after 52 weeks of Covishield vaccination. These findings offer crucial information on the safety of the vaccine regarding these particular cardiac events.

## Conclusion:

Data shows that post-vaccination myocarditis is typically mild and transient, with most cases resolving swiftly through conservative management. Therefore, it is unlikely that this vaccine will induce severe arrhythmias or life-threatening cardiac events in the general population.

## Limitation of study:

The limitation of our study is the small sample size, which reduces its applicability to a larger population. Large prospective population studies with longitudinal follow-up are needed to understand the long-term effects of the Covishield vaccine for various age and sex subsets. Secondly, we were only able to examine Lead II ECG due to the limitation of our Power Lab data collection equipment. All twelve leads of the ECG should be taken into consideration in further studies where possible.

## Financial support and sponsorships:

Nil.

## Figures and Tables

**Table 1:** Anthropometric parameters of participants

**Parameters**	**Male ( mean ± SD)**	**Female(mean ± SD)**
Age (years)	66.75±3.025	63.12±2.054
Body weight (kg)	70.25±2.375	54.036±2.885
Height (meter)	1.702±0.228	1.560±0.028
BMI (kg/m^2^)	24.236±1.480	21.418±1.577
Mean ± SD, SD, standard deviation; BMI, body mass index.

**Table 2:** Haemodynamic parameters and ECG intervals of the participants

**Parameter**	**Male ( mean ± SD)**	**Female ( mean ± SD)**
Heart Rates(Beats/minutes)	80.325 ± 8.096	84.906 ± 10.225
SBP (mm Hg)	134.1 ± 2.7	128.88 ± 2.764
DBP (mmHg)	85.55 ± 1.995	77.92 ± 6.176
RR(Second)	0.763 ± 0.077	0.721 ± 0.085
PR interval(Seconds)	0.159 ± 0.018	0.153 ± 0.024
P duration(Second)	0.96 ± 0.011	0.090 ± 0.024
QRS interval(Seconds)	0.075 ± 0.011	0.063 ± 0.010
QT interval(Seconds)	0.318 ± 0.014	0.317 ± 0.025
QTc (Seconds)	0.366 ± 0.021	0.375 ± 0.025
JT interval (Seconds)	0.242 ± 0.014	0.254 ± 0.022
T-peak T-end interval(Seconds)	0.052 ± 0.006	0.055 ± 0.008
Mean ± SD, SD, standard deviation;
SBP, systolic blood pressure; DBP, diastolic blood pressure;
HR, heart rate.

**Table 3:** ECG wave amplitudes of the participants

**Parameter**	**Male ( mean ± SD)**	**Female ( mean ± SD)**
P (mV)	0.129 ± 0.027	0.121 ± 0.032
Q (mV)	-0.081 ± 0.046	-0.073 ± 0.042
R (mV)	0.918 ± 0.228	1.035 ± 0.418
S (mV)	-0.144 ± 0.117	-0.126 ± 0.084
T (mV)	0.219 ± 0.118	0.181 ± 0.076
Mean ± SD, SD, standard deviation
